# Data for effects of lanthanum complex on the thermo-oxidative aging of natural rubber

**DOI:** 10.1016/j.dib.2015.10.032

**Published:** 2015-11-06

**Authors:** Wei Zheng, Li Liu, Xiuying Zhao, Jingwei He, Ao Wang, Tung W. Chan, Sizhu Wu

**Affiliations:** a Beijing Engineering Research Center of Advanced Elastomers, Beijing University of Chemical Technology, Beijing 100029, China; b Engineering Research Center of Elastomer Materials Energy Conservation and Resources, Ministry of Education, Beijing University of Chemical Technology, Beijing 100029, China; c Department of Materials Science and Engineering, Virginia Polytechnic Institute and State University, Blacksburg, VA 24061, USA

## Abstract

Novel mixed antioxidants composed of antioxidant IPPD and lanthanum (La) complex were added as a filler to form natural rubber (NR) composites. By mechanical testing, Fourier transform infrared spectroscopy with attenuated total reflectance (FTIR-ATR) and thermogravimetric analysis (TGA), a string of data, including the mechanical properties, the variation of internal groups and the thermal and thermo-oxidative decompositions of NR, was presented in this data article. The data accompanying its research article [1] studied the thermo-oxidative aging properties of NR in detail. The density function theoretical (DFT) calculations were also used as an assistant to study the thermo-oxidative aging mechanism of NR. The data revealed that this new rare-earth antioxidant could indeed enhance the thermo-oxidative aging resistance of NR, which is associated with its different function mechanism from that of the pure antioxidant IPPD.

**Specifications table**

**Table:** 

Subject area	*Materials science*
More specific subject area	*Rare earth complex as antioxidant*
Type of data	*Figure, spectra, table*
How data was acquired	*An electronic tensile machine (Shenzhen SANS Test Machine Co., Ltd., China); FTIR-ATR (a Nicolet 8700 FTIR spectrometer, Thermo Fisher Scientific Inc., USA); TGA (a TGA/Differential Scanning Calorimetry (DSC) calorimeter, Mettler-Toledo Co., Switzerland); Gaussian 09 suit of program*
Data format	*Raw, analyzed*
Experimental factors	*After being mixed on a two-roll mill, the compounds were cured in a 25* *T hot compression mold at 143* *°C for t_90_ to gain NR composites.*
Experimental features	*Analyze the mechanical properties, microstructure, thermal and thermo-oxidative decomposition of NR composites before and after thermal oxidation exposures.*
Data source location	*Beijing University of Chemical Technology, Beijing, China*
Data accessibility	*The data is with this article.*

**Value of the data**

• These data provides a complete set of methods for other scientists to study the thermo-oxidative aging properties of materials.
• These data proposes a theoretical method for investigating the function mechanism of different antioxidants.
• These data has important significance for the exploitation of rubbers with high resistance to thermo-oxidative aging.

## 1 Data

Data presented in this article was used to investigate the different protective effects on the thermo-oxidative aging properties of two formulations of NR – one with the antioxidant IPPD and the other with the mixed antioxidants composed of antioxidant IPPD and lanthanum (La) complex. In addition, the data about theoretical calculations was also used as an assistant to investigate the different function mechanisms of the antioxidant IPPD and La complex.

## 2 Experimental design, materials and methods

### 2.1 Materials

**Fig. 1:**
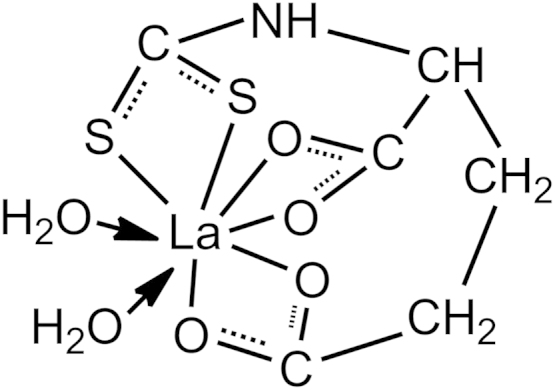
The structural formula of DAGLa.

The structural formula of dithio-aminomethyl-glutamic acid lanthanum (DAGLa) was shown in Fig. 1. The base formulation of the two NR composites was described as follows (phr): RSS, 70; E-SBR 1500, 30; zinc oxide, 5; stearic acid, 2; silica-VN3, 40; Si69, 4; Carbon black 234, 5; sulfur, 1.8; accelerator CZ, 1.5.

In addition, 3 phr of antioxidant IPPD was added into the base formulation to prepare Sample I, while 3 phr of mixed antioxidants (antioxidant IPPD, 2.25; DAGLa, 0.75) were added to the base formulation to prepare Sample II.

### 2.2 Experimental design and methods

#### 2.2.1 Data of mechanical properties

Table 1 shows the mechanical properties measured before and after 120 h of thermal oxidation exposure at 80 °C. The mechanical tests were carried out according to Chinese standard GB/T 528-2009. After aging, the retained ratios of tensile strength for the two composites are 88.13% and 91.74%, and the retained ratios of elongation at break are 71.5% and 76.84%.

#### 2.2.2 FTIR-ATR spectra

**Fig. 2:**
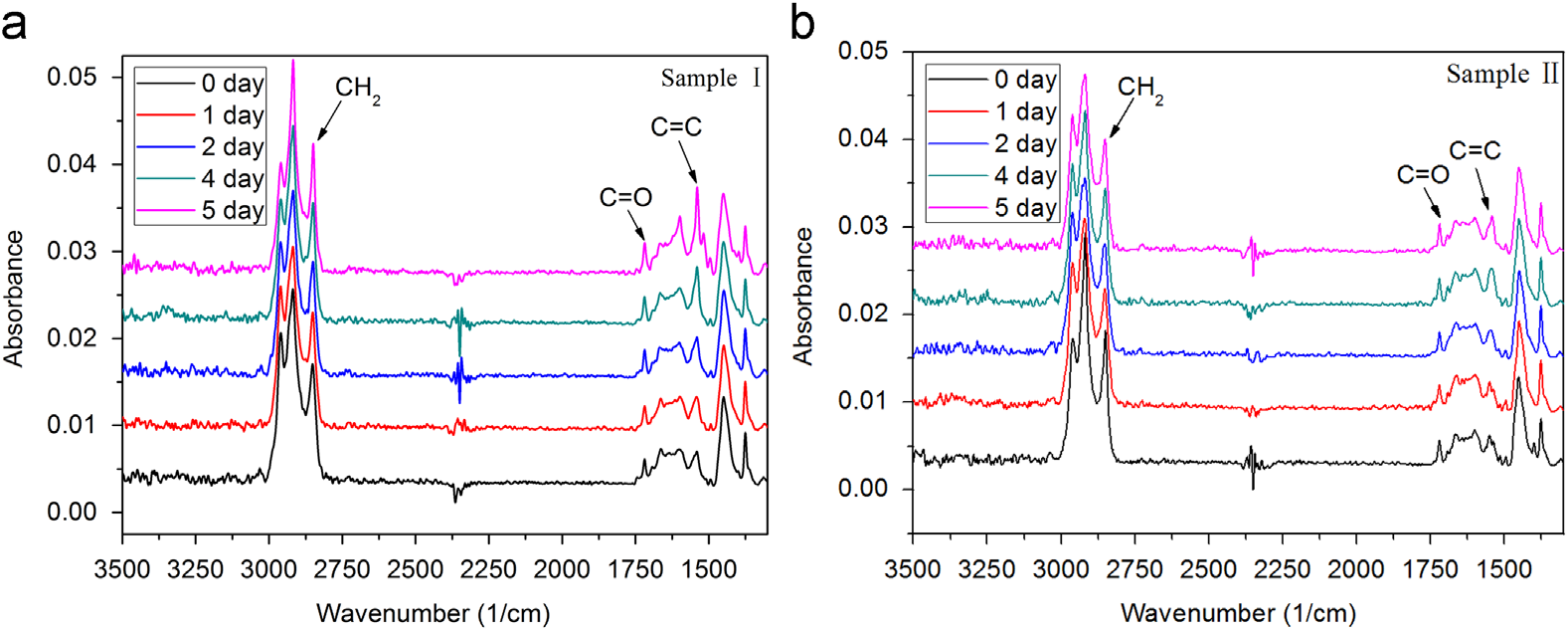
FTIR-ATR spectra of two NR composites aged at 80 °C: (a) Sample I; (b) Sample II.

The FTIR spectra were acquired by using the attenuated total reflection (ATR) technique in the wavenumber range of 4000–600 cm^−1^ with scan times of 32 at 8 cm^−1^ resolution. As is shown in Fig. 2, the absorption bands for C=O stretching vibration at 1737 cm^−1^ and C=C stretching vibration at 1538 cm^−1^ increase in intensity with increasing aging time, especially for Sample I, indicating the generation of ketone, ester, etc., and the scission of the NR main chain during the thermo-oxidative aging.

**Fig. 3:**
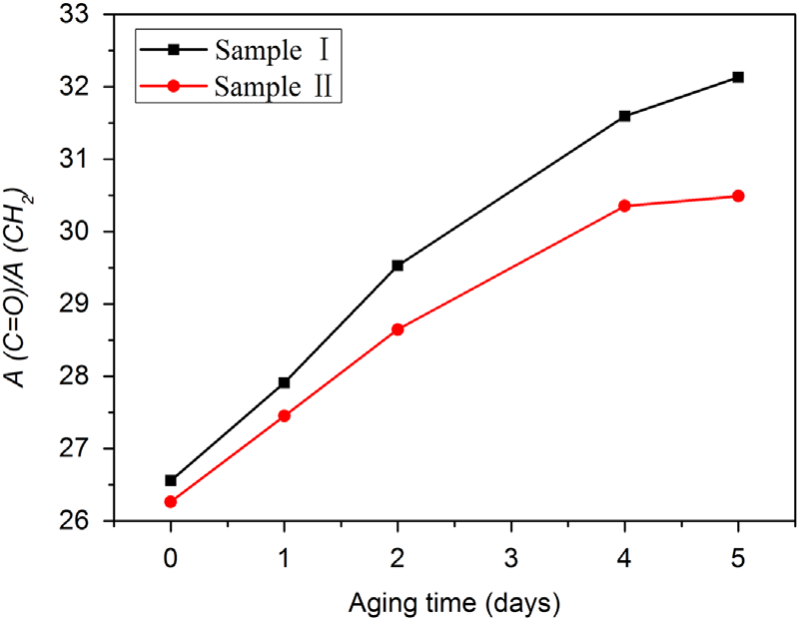
Absorbance ratio (A(C=O)/A(CH2)) as a function of aging time for two NR composites aged at 80 °C.

Previous studies [2] showed that the absorption bands at 2848 cm^-1^ (CH_2_ symmetric stretching vibration) is unaffected by thermo-oxidative aging. Thus, the ratio of the absorbance of C=O to that of CH_2_ (A(C=O)/A(CH_2_)) can quantitatively reflect the thermo-oxidative degree of NR composites, which is shown in Fig. 3.

#### 2.2.3 Thermal and thermo-oxidative decomposition data

**Fig. 4:**
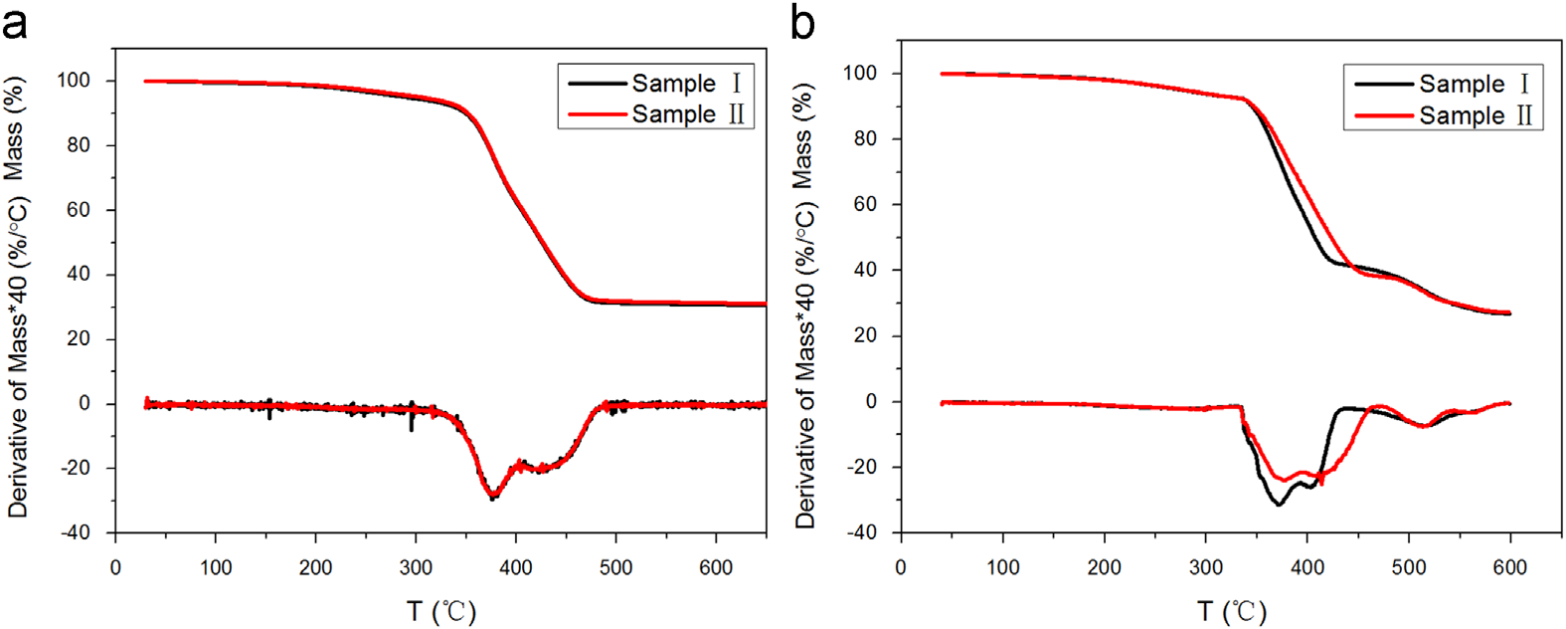
TG–DTG curves in different atmosphere of two NR composites: (a) in N_2_; (b) in air.

The thermogravimetry (TG) and corresponding differential thermogravimetry (DTG) curves of Sample I and II at heating rate of 10 K/min in both nitrogen (N_2_) and air atmospheres are presented in Fig. 4 to study the thermal and thermo-oxidative decompositions of NR. And the thermal and thermo-oxidative decomposition characteristics of the two composites are presented in Table 2.

In the N_2_ atmosphere, the thermal decomposition of NR is a one-stage reaction. The mixed antioxidants do not make shape changes to the TG and DTG curves; however, data in Table 2 shows the thermal decomposition temperatures of Sample II are slightly higher than those of Sample I. The mechanism of the thermal decomposition of NR is a free radical chain reaction [3]. Random scissions of the NR main chain generate various hydrocarbon radicals. The nearly overlapping curves show DAGLa exhibits poor abilities to react with hydrocarbon radicals and does not have obvious effect to improve the thermal decomposition of NR.

In the air atmosphere, the DTG curves exhibit two peaks; therefore, the thermo-oxidative degradation reaction can be considered as a two-stage reaction. As the weight loss at about 350 to 425 °C in the air atmosphere is lower than that in the N_2_ atmosphere, the first decomposition stage is considered to be the thermo-oxidative decomposition of NR chain. And the second decomposition stage is the further decomposition of thermo-oxidative aging products. Data in Table 2 shows an increase in thermo-oxidative decomposition temperature from Sample I to Sample II at different decomposition levels, indicating that Sample II has higher thermo-oxidative stability than Sample I.

#### 2.2.4 Kinetic data

The non-isothermal, isoconversional Flynn–Wall–Ozawa (FWO) method analyzed by TGA curves was proposed to determine the kinetic data of thermo-oxidative degradation. Each sample weighing about 7–10 mg was heated from 30 to 600 °C at different heating rates (5 K/min, 10 K/min, 20 K/min, and 30 K/min) under an air flow of 50 mL/min. This method is based on the Doyle [4] approximation for heterogeneous chemical reactions:

(1) $$\log\;\beta =log\left(\frac{A\Delta E_{a}}{R\cdot g(\alpha )}\right)-2.315-0.4567\frac{\Delta E_{a}}{RT}$$

where *A* is a pre-exponential factor, $\Delta E_{a}$ is the activation energy, *R* is the gas constant, *α* is the fractional mass loss, and *g(α)* is a conversional function.

The FWO principle is based on the assumption that the reaction rate at a given fractional mass loss (*α*) is only a function of the temperature. Therefore, for different heating rates (*β*) at a constant *α*, *E_a_* can be estimated from the slope of the straight line obtained by plotting *logβ* versus *T*^*−1*^.

**Fig. 5:**
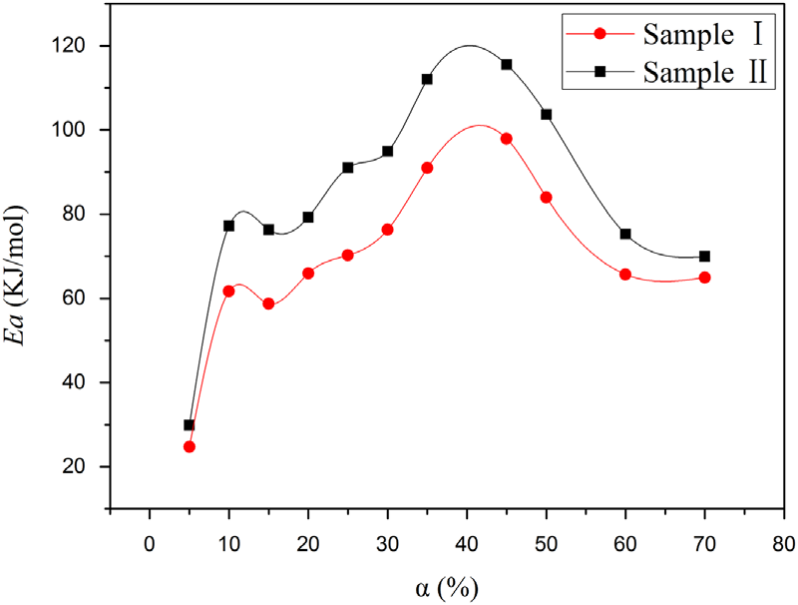
Plots of *E_a_* versus fractional mass loss (*α*) determined by FWO analysis.

Fig. 5 shows plots of *E_a_* versus *α* according to the FWO method (Eq. (1)). For an ordinary thermo-oxidative degradation process, the changes of *E_a_* can be divided into three stages [5]. In the first stage, *E_a_* increases with *α* until *α* reaches 10%, corresponding to the initiation of the chain. In the second stage, when *α* is from 10% to 15%, *E_a_* decreases with *α* because of the autocatalytic oxidation process during thermo-oxidative degradation [2], [5]. In the final stage, at *α* higher than 15%, *E_a_* increases with *α* again, reaches a maximum at *α* of nearly 40%, and then decreases with *α*. This final stage is associated with the decomposition of thermo-oxidative products and the intensification of the thermo-oxidative degradation process. Furthermore, the activation energies required for thermo-oxidative degradation are higher for Sample II than for Sample I.

#### 2.2.5 Different function mechanisms of two kinds of antioxidants

All these molecular structures were constructed by GaussView. Density Function Theoretical (DFT) calculations were performed using the Gaussian 09 suit of program [6]. Geometry optimizations for all the molecules were simulated using B3LYP hybrid method and 6-31 G(d) basis set to gain the energy of the molecules. The dissociation energy (*E_d_*) was calculated by the following equation:

(2) $$E_{d}=E_{A}+E_{H}-E_{AH}$$

where *E*_*A*_ and *E*_*H*_ are the energies of two free radicals generated by the hemolysis of the covalent bond, and *E*_*AH*_ is the energy of compound AH.

**Fig. 6:**
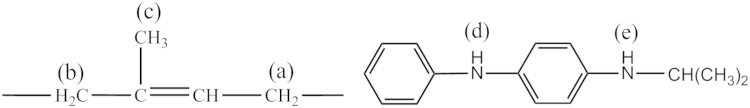
The position (a), (b) and (c) of C–H bonds in NR and the position (d) and (e) of antioxidant IPPD.

The principal mechanism of the thermo-oxidative aging of NR involves an autocatalytic, free radical chain reaction [3]. The first step is the initiation of the chain. As a typical amine antioxidant, antioxidant IPPD, whose structure is shown in Fig. 6, has an active hydrogen atom (H) in imino group (d). With the less dissociation energy of 328.02 KJ/mol (Table 3) than C–H bonds in NR chain, this H can compete effectively with that in NR chain to terminate the active free radicals and generate free radicals with low activity. And this H also makes it easy for antioxidant IPPD to react with oxygen (O_2_) directly.

La has the electron shell structure as 5d^1^6s^2^ and the valence state of ion as +3. The full empty 4d orbit and long atom radius result in strong coordination abilities and large coordination numbers, which exceed 6, normally are 7, 8, 9, and 10. Compared with IPPD (molecular weight=226), which has only one active position to react with one free radical, the same mass of DAGLa (molecular weight=395) can scavenge more oxy radicals [8]. In addition, the existence of the thioether bonds in DAGLa is useful to decompose the hydroperoxide [2]. These mean that the DAGLa is more effective than antioxidant IPPD to enhance the thermo-oxidative stability of NR.

## Figures and Tables

**Table 1:** Mechanical properties of two NR composites before and after aging.

Samples	Condition	Tensile strength (TS) (MPa)	Elongation at break (Eb) (%)	Tensile stress at 100% (Se) (MPa)	Tensile stress at 300% (Se) (MPa)
I	Before aging	23.50±0.59	591.04±10.78	2.34±0.04	7.91±0.16
80 °C×120 h aging	20.71±0.96	422.58±16.49	3.60±0.15	12.72±0.56
					
II	Before aging	23.72±0.53	568.89±13.02	2.33±0.11	8.38±0.61
80 °C×120 h aging	21.76±0.78	437.13±4.70	3.45±0.12	12.84±0.47

**Table 2:** Thermal and thermo-oxidative decomposition characteristics of two NR composites at heating rate of 10 K/min in N_2_ and air, where *T*_*max*_ refers to the temperature when the DTG curve reaches the maximum value.

Samples	Atmosphere	*T* /°C corresponding to certain mass loss /%				*T*_*max*_ /°C
5	10	15	20
I	N_2_	291.83	350.00	363.33	371.83	380.83
Air	276.94	345.68	355.23	363.51	371.74
II	N_2_	305.50	351.83	364.33	370.83	381.17
Air	279.53	347.10	360.11	369.55	376.59

**Table 3:** The dissociation energy values of different C–H bonds in NR and different N–H bonds in antioxidant IPPD.

Position	Dissociation energy(exp) [7]/KJ/mol	Dissociation energy(sim)/KJ/mol
(a)	322.61	378.89
(b)	335.58	387.03
(c)	351.48	389.10
(d)	–	328.02
(e)	–	484.39

Ps: The position (a), (b) and (c) of C-H bonds and the position (d) and (e) of antioxidant IPPD are as follows.
